# Optimization of Big Data Scheduling in Social Networks

**DOI:** 10.3390/e21090902

**Published:** 2019-09-17

**Authors:** Weina Fu, Shuai Liu, Gautam Srivastava

**Affiliations:** 1 College of Information Science and Engineering, Hunan Normal University, Changsha 410081, China; fuwn@imau.edu.cn (W.F.); liushuai@hunnu.edu.cn (S.L.); 2 Hunan Provincial Key Laboratory of Intelligent Computer and Language Information Processing, Hunan Normal University, Changsha 410081, China; 3 College of Computer Science, Inner Mongolia University, Hohhot 010012, China; 4 Department of Mathematics and Computer Science, Brandon University, Brandon, MB R7A 6A9, Canada; 5 Research Center for Interneural Computing, China Medical University, Taichung 40402, Taiwan

**Keywords:** big data, database design, entropy, information transfer, social networks, information security, scheduling, task volume, classification, optimization

## Abstract

In social network big data scheduling, it is easy for target data to conflict in the same data node. Of the different kinds of entropy measures, this paper focuses on the optimization of target entropy. Therefore, this paper presents an optimized method for the scheduling of big data in social networks and also takes into account each task’s amount of data communication during target data transmission to construct a big data scheduling model. Firstly, the task scheduling model is constructed to solve the problem of conflicting target data in the same data node. Next, the necessary conditions for the scheduling of tasks are analyzed. Then, the a periodic task distribution function is calculated. Finally, tasks are scheduled based on the minimum product of the corresponding resource level and the minimum execution time of each task is calculated. Experimental results show that our optimized scheduling model quickly optimizes the scheduling of social network data and solves the problem of strong data collision.

## 1. Introduction

Social networking services delivered via the Internet have come to permeate every corner of people’s lives [1], including business, academia, entertainment and dating, and their commercial and academic value are gradually increasing. With the development of “Web 2.0” technologies, the amount of data on the Internet has grown explosively [2,3]. However, more of this data is unstructured or semi-structured, rather than structured [4,5,6]. Because of this explosion of data, data sources are no longer singular and such data requires processing to yield meaningful insights [7,8]. Target data can be obtained from massive amounts of social network data using a reasonable scheduling method [9,10] and the problem of realizing accurate and efficient scheduling of massive data in social networks is one that requires analysis. This issue has also received the attention of relevant technical personnel. Finally, we have also seen optimization efforts for cloud systems. Persico et al. propose reproducible methodology to assess performance over cloud platforms [11,12]. Due to the breadth of the research thus far, we propose a new, optimized method for big data scheduling in social networks to provide a theoretical basis for the field of social network data management.

### Related Work

When constructing a big data scheduling model in social networks, the task of data communication in transmission of target data is not considered comprehensively. Therefore, data scheduling models of big data in social networks is prone to generate conflict of data at nodes. Dinh et al. studied the data scheduling and admission control of Backscatter Sensor Network (BSN), then introduced a system model and a mechanism to solve the data acquisition and scheduling problems in BSN [13]. In their research, both an optimization scheme based on framework of Markov decision process and an enhanced learning algorithm based on linear function approximation are proposed to find the optimal data scheduling strategy for the gateway.

Yang applies Software-defined networks (SDN) to data center networks and proposes a real-time traffic scheduling method based on SDN in Reference [14]. In SDN network architecture, the performance parameters of the underlying network (current link available bandwidth, link delay, packet loss rate, etc.) can be obtained through the interaction of Open Flow messages between the controller and the switch. When calculating routing, these real-time parameters are added to the routing computation metrics. The acquired real-time link delay and packet loss rate are taken as the routing computation metrics of rat stream transmission and the available link bandwidth is taken as the routing computation metrics of elephant stream transmission. Then, the hard threshold of transmission delay is added as a new limitation and the objective function is established to minimize the network routing metrics and complete real-time traffic data scheduling.

Dong et al. analyze the multi-path forwarding strategy of Named data networking (NDN) please define and the combination between NDN and SDN in Reference [15]. A traffic scheduling method based on centralized control for data network is proposed by introducing the centralized control mechanism to optimize the traffic forwarding in NDN. The overall architecture and traffic model are designed according to the characteristics of NDN and only appropriate forwarding strategies for hot content requests is deployed in some nodes. Experiment results show that it can effectively optimize the global traffic scheduling and reduce the communication overhead between the controller and the nodes.

Khabbaz et al. present a time-based process scheduling method in Reference [16]. In addition to reducing average process completion time (FCT), their proposed method aims to reduce probability of both deadline mismatches and blocking, thereby improving average application throughput. They establish an analytical queuing model for capturing the network dynamics of data centers and evaluating the performance of data centers running under Data Asset Framework (DAF).

Hou et al. allocate various types of network resources to users for “QoS fairness” [17]. It aims to balance the quality of service and fairness in WLAN by allocating various types of network resources to users. To this end, the user’s Quality of Service (QoS) requirements are first transformed into multi-resource requirements and the dominant mechanism of resource fairness is applied to allocate network resources for each user, which is the foundation for data scheduling.

Céline studies the establishment of a forwarding tree for collecting and aggregating sensing data in the network under the actual physical interference model in Reference [18]. The acquisition tree construction and link scheduling are solved jointly, with a low computational complexity. Their aim is to collect data on the receiver with minimum delay and less transmission and coordinate the transmission between network links to control interference, so as to facilitate the implementation of data scheduling.

Based on the above methods, our paper is the first to calculate the loading task of big data communication according to the transmission state of target data in the server. Then, we introduce the task scheduling model of big data applied to social networks to solve the problem of node data conflicts. We achieve the optimal scheduling of big data classification in social networks by using the minimum product value. We show an improved speed of data scheduling in social networks and the ability to solve data conflicts effectively.

## 2. Entropy in Social Networks

The robustness of any defined system can be found by calculating its entropy, which is defined as the system’s degree of disorder or, more specifically, the number of microstates the system possesses. Finding the number of these states can be accomplished by the well-known Boltzmann equation given in Equation (1).

(1) $$S=k_{b}\ln W$$

where *S* is the entropy, k${}_{b}$ is Boltzmann’s constant and W is the number of microstates. Using statistical mechanics, an expression of entropy in terms of probability distribution seems more plausible and practically more versatile, as most statistical problems, such as the network problem, lead to a distribution function of the number of microstates rather than an actual number. Entropy is described through probability distribution in Equation (2).

(2) $$S=-\sum_{k}P(k)\ln P(k)\;$$

where P(k) is the probability that the system is in state *k*. Besides basic entropy, there are three forms of calculated entropy in an information transfer domain: (i) search entropy, (ii) access entropy and (iii) target entropy. In this paper, we focus on access and target entropy. The interpretation of “entropy” as used in social network analysis is controversial. One interpretation of entropy, the degree of robustness of the network, leads to a profound philosophical question—what does “robustness” mean or refer to in a social network? “Robustness”, for social networks, implies that social links are dynamic and allowed to be changed with and without restrictions or constraints. This indeed is applicable to all social networks. Some social networks are more dynamic as the restrictions on changes in social links are either minimal or non-existent, such as that of Facebook friends or LinkedIn connections. On the other hand, there also exist constrained social networks where social links are rigid and cannot experience change, such as in the real-life social networks of close families and organized crime syndicates. These links are almost completely rigid and unchanging over time; consequently, social networks built on them possess a small number of possible configurations (shapes). In summary, social networks can be classified into one of two extremes—unconstrained, very robust social networks, which have very low entropy and constrained, very non-robust social networks, which have very high entropy. A network is said to be in equilibrium, a state of no driving force potential for the network to change its configuration, when it has the maximum entropy for a given number of nodes. In this work, we focus on unconstrained robust social networks and try to optimize their target entropies using mass data scheduling techniques.

## 3. Material Method

### 3.1. Scheduling Model

#### 3.1.1. Partition Calculation for Scheduling Task Volume

First, the amount of massive data scheduling for social networks is calculated and is expanded in a partitioned computing manner. The overall task of scheduling big data in a social network is divided into several subtasks, which are defined as $u={\left[u\left(0\right),u\left(1\right),\cdots ,u\left(e-1\right)\right]}^{k}$. and the condition a∼g(u,μ) is satisfied for the arrangement state. Next, the total number of scheduled tasks is calculated by Equation (3)

(3) $$\hat{\mu }=arc\min_{\begin{array}{c} -\\ q\in \varepsilon \end{array}}\sum_{\mu =1}^{+\infty }H\left(\hat{\mu },\mu \right)g\left(\mu |u\right)$$

where $H(\hat{\mu },\mu )$ represents the cost function of the task, g(μ|u) represents the distribution probability of the subtask and ε is the set of all scheduling subtasks, which is calculated by Equation (4)

(4) $$\varepsilon =\left\{\hat{\mu }|u_{1}+2u_{r}\leq \hat{\mu }\leq S\right\}$$

where *S* is the upper limit on the amount of tasks and the corresponding cost function is calculated by Equation (5)

(5) $$H\left(\hat{\mu },\mu \right)={\left(\hat{\mu }-\mu \right)}^{2}$$

Equation (6) is obtained by substituting Equation (5) into Equation (3)

(6) $$\hat{\mu }=\sum_{\mu =1}^{\mu }\mu \hat{\mu }\left(\mu |\mu \right)$$

where $\hat{\mu }$ represents the average time when the data is scheduled. Assuming the scheduling time satisfies the condition $\sum_{\mu =1}^{\mu }\hat{\mu }\left(\mu |u\right)=1$, the estimation function is defined as Equation (7)

(7) $$H\left(\hat{\mu },\mu \right)=\left|\hat{\mu }-\mu \right|$$

Then, substitute Equation (7) into the task amount calculation formula and obtain Equation (8)

(8) $$\hat{\mu }=arc\min_{\mu }\hat{\mu }\left(\sum_{\mu =1}^{}\mu \left(\mu |\mu \right)-\sum_{\mu =\hat{\mu }}^{\mu }\mu \left(\mu |u\right)\right)$$

Finally, the number of subtasks in scheduling is found by Equation (9).

(9) $$\mu =arc\underline{\max}q\left(\bar{\mu }|u\right)$$

The above method is used to obtain the subtask for massive data scheduling task of a social network and massive data scheduling model of a social network is constructed based on the data foundation.

#### 3.1.2. Construction of Massive Data Scheduling Model for Social Networks

During the process of scheduling big data in social networks, the existence of a large amount of interfering information and the randomness of data nodes lead to conflicts in data nodes of the scheduling model, which seriously affects accuracy and efficiency [19,20,21]. Therefore, the task quantity of big data communication is calculated based on the transmission state of target data in the server. In addition, an optimized big data scheduling model is constructed to schedule massive data in a social network to avoid target data conflicting in the same data node.

*z* is defined to indicate the amount of data sent by data nodes per unit of time and γ indicates the fuzzy value of transmission efficiency for data nodes, where γ∈[0,1]. The calculation method of the data efficiency increment coefficient of the data node for a scheduling model is as follows.

(10) γ=v(z)

A mapping relationship between the massive data scheduling task of a social network and data nodes is constructed based on the above equation. $\gamma =\left\{\gamma_{1},\gamma_{2},\cdots ,\gamma_{q}\right\}$ is defined to represent a data set consisting of all scheduled task quantities in the mapping relationship, where $\gamma_{i}$ represents the data sent by the *i*-th data node. Target data sent by the data node *T* to request is defined as $j_{i}$, where $j=\left\{\lambda_{1}j_{1},\lambda_{2}j_{2},\cdots ,\lambda_{q}j_{q}\right\}$ and condition $U_{i},j_{i}\in \left[0,1\right]$ is satisfied. The priority level of big data scheduling is $\lambda_{i}$. During execution of data scheduling tasks, there is a constraint on the amount of data sent by data nodes [22] and calculation method of data volume is as shown in Equation (11).

(11) $$h\left(\gamma_{1},\gamma_{2},\cdots ,\gamma_{q}\right)=1$$

The efficiency of scheduling tasks is calculated by Equation (12).

(12) $$\varphi =\left|\gamma -j\right|=\sqrt{{\left(\gamma_{1}-\lambda_{1}j_{1}\right)}^{2}+{\left(\gamma_{2}-\lambda_{2}j_{2}\right)}^{2}+\cdots +{\left(\gamma_{q}-\lambda_{q}j_{q}\right)}^{2}}$$

where φ indicates the efficiency of scheduling tasks. The scheduling tasks are efficient when the correlation between γ and *j* is small.

The massive data scheduling model for social networks is constructed in Equation (13)

(13) $$\min\varphi =\sqrt{{\left(\gamma_{1}-\lambda_{1}j_{1}\right)}^{2}+{\left(\gamma_{2}-\lambda_{2}j_{2}\right)}^{2}+\cdots +{\left(\gamma_{q}-\lambda_{q}j_{q}\right)}^{2}}$$

where $\gamma_{i},j_{i},\lambda_{i}\in \left[0,1\right]$, i=1,2,⋯,q.

The model constructed above takes into account each task’s amount of data communication during transmission of target data. The big data scheduling model is constructed according to the quantity of tasks, which effectively solves the problem of target data conflicting in the same data node.

In big data classification optimization scheduling, it is assumed that the task interval of periodic tasks is *A*, which is understood as the total time taken to complete the current instance and the next instance of a classification optimization scheduling task [23]. If there are multiple tasks and they are selectively executed, the periodic function selects the task with the smallest task cycle to perform classification optimization scheduling first. If there are *m* tasks in a periodic task set, the constraints of scalable scheduling for big data cycle tasks are obtained by Equations (14)–(17)

(14) $$B_{i}\left(A\right)=\sum_{i=1}^{m}b_{i}\left[\frac{A}{q_{i}}\right]$$

(15) $$d_{i}\left(A\right)=\frac{B_{i}\left(A\right)}{A}$$

(16) $$D_{i}=\min_{0\leq A\leq q_{i}}\left(A\right)$$

(17) $$D=\max_{i=1,2,m}\left\{D_{i}\right\}$$

where $B_{i}\left(A\right)$ represents weight of the task interval *A*. The periodic big data classification optimization scheduling task set is set to $q_{i}$. $b_{i}$, which denotes its optimization scheduling period. Periodic task classification optimizes a constraint D≤1 of the scheduling.

In classification and optimization of scheduling of big data, the data does not have periodic tasks and it is impossible to comprehensively study the randomness and contingency of its activities. The stopping time of each periodic task is also difficult to determine [24,25]. Therefore, we use stochastic process theory to describe the occurrence process that is an aperiodic task, and to calculate the distribution function and mathematical expectation that do not rely on the task being periodic.

In big data classification optimization scheduling, aperiodic tasks will be combined with the parameter ϕA into a Poisson distribution in interval [0,A]. The likelihood function $D\left(y_{1},y_{2},\ldots ,y_{m},\phi \right)$ is obtained by Equation (18)

(18) $$D\left(y_{1},y_{2},\ldots ,y_{m},\phi \right)=\frac{\sum_{\phi_{i}=1}^{m}y_{i}}{y_{1},y_{2},\ldots ,y_{m}}d^{-m\phi }$$

where the mathematical expectation of interval [0,A] is set to ϕA and ϕA is also understood as the number of times the mathematical expectation occurs in unit time expressed in ϕ. *y* represents a variable of classification optimization scheduling. *d* represents a scheduling time factor.

Construction of the scheduling model is completed by Equation (19). The model considers the task amount of data communication when target data is transmitted [26], which effectively solves the problem of target data conflicts in the same data node.

(19) $$\left\{\begin{array}{c} \phi =\frac{1}{m}\sum_{i=1}^{m}y_{i}=\bar{y}=d\left(y\right)\\ \min\varphi =\sqrt{{\left(\gamma_{1}-\lambda_{1}j_{1}\right)}^{2}+{\left(\gamma_{2}-\lambda_{2}j_{2}\right)}^{2}+\cdots +{\left(\gamma_{q}-\lambda_{q}j_{q}\right)}^{2}} \end{array}\right.$$

### 3.2. Optimized Implementation of Massive Data Scheduling Model in Social Networks

#### 3.2.1. Analysis of Schedulable Conditions for Massive Data Classification Tasks in Social Networks

According to the above calculation, when big data classification optimization scheduling is performed, the task *A* is a periodic task with completion time $c_{A}$, preparation time $e_{A}$, running time $r_{A}$ and stop time $w_{A}$. When big data classification optimization scheduling is performed, *E* is a non-periodic task with task completion time $c_{E}$ and preparation time $e_{E}$. The scheduling stop time for aperiodic tasks is given by *w*.

In big data classification optimization scheduling, we define *n* to be the number of processors and *m* to be the number of periodic tasks. $d_{m}$ indicates the execution time of the classification optimization scheduling, $A_{m}$ indicates the stop time of the task classification optimization scheduling. *g* is the number of aperiodic tasks, $d_{Sg}$ is the task execution time and $A_{Sg}$ is the average stop time of the task. From these definitions, the schedulable constraint for big data classification tasks is obtained in Equation (20).

(20) $$n-\left[\frac{d_{1}}{A_{1}}+\frac{d_{2}}{A_{2}}+\ldots +\frac{d_{m}}{A_{m}}\right]\geq \left[\frac{d_{S1}}{A_{S1}}+\frac{d_{S2}}{A_{S2}}+\ldots +\frac{d_{Sg}}{A_{Sg}}\right]$$

In the optimization scheduling of big data classification, the main ideas for the equalization of data tasks are as follows—When the processor is isomorphic, the number of classification optimization scheduling task types is set to *N*, where N≥3. In addition, for the task $A_{i}$ in the task type *j*, j≤N. Then, the following relationship is obtained in Equation (21).

(21) $$\frac{1}{2^{J+1}}-1\leq \frac{d_{i}}{A_{i}}\leq \frac{1}{2^{J+1}}-1$$

When the big data scheduling performs classification optimization, the type of task $A_{i}$ is *j*.

Based on the above calculations, the optimization classification of massive data scheduling is optimized. The detailed process is as follows. Firstly, the constraint conditions of the scalable scheduling for periodic tasks and the distribution function of computational periodic tasks are analyzed. Then, a massive data classification optimization scheduling model in social networks is established to lay the foundation for later optimization.

#### 3.2.2. Big Data Classification and Optimization Scheduling Method in Social Networks

Based on the model in the previous section, when big data scheduling performs classification optimization, the execution value of each task in all social network resources is calculated. This means that each classification optimizes for a product of the corresponding resource level and the minimum execution time of scheduling task [27,28]. Then, the minimum value of this product is obtained and the optimization scheduling of massive data classification in a social network is completed.

It is assumed that the number of *m* classification optimization scheduling tasks is $X=\left\{x_{1},x_{2},\ldots ,x_{m}\right\}$ and the number of *n* social network resources is $Y=\left\{y_{1},y_{2},\ldots ,y_{n}\right\}$. Then, we iterate through the following steps until all the sets are empty:

1. In classification optimization of big data scheduling, the optimal minimum time (min time) ($x_{i}$ to $y_{1},y_{2},\ldots ,y_{n}$) is calculated by Equation (22)
  (22) $$x_{i}A_{\min}\left(i\right)=Min\left(\min time\left(x_{i}\right)y_{1},y_{2},\ldots y_{n}\right)$$
  where $x_{i}A_{\min}\left(i\right)$ represents the minimum time consumption of big data classification optimization scheduling.
2. When classifying and optimizing big data, Equation (23) is used to obtain the two-dimensional array $com_{sx}\left[i,j\right]$.
  (23) $$com_{sx}\left[i,j\right]=x_{i}A_{\min}\left(i\right)\times \mathrm{Resource}\;\mathrm{level}$$
3. When big data classification optimization scheduling is performed, $com_{sx}\left[i,j\right]$ is sorted to obtain a minimum $com_{sx}^{}\left[i,j\right]$.
4. $x_{i}$ is dispatched to $y_{j}$ when big data classification optimization scheduling.

The principle of big data scheduling optimization in social networks is described using the above calculations, completing the optimization of big data scheduling.

## 4. Results

To verify the effectiveness and superiority of our proposed method, we carry out optimization analysis of big social network data scheduling using the ns-2.34 network simulation platform.

### 4.1. Throughput Analysis

Our simulation includes three network interfaces referred to as path A, path B and path C, with bandwidths of 0.35 MB/s, 0.65 MB/s and 0.95 MB/s, respectively; 1.45 MB/s is the bandwidth for the application layer data of the sender.

**Figure 1:**
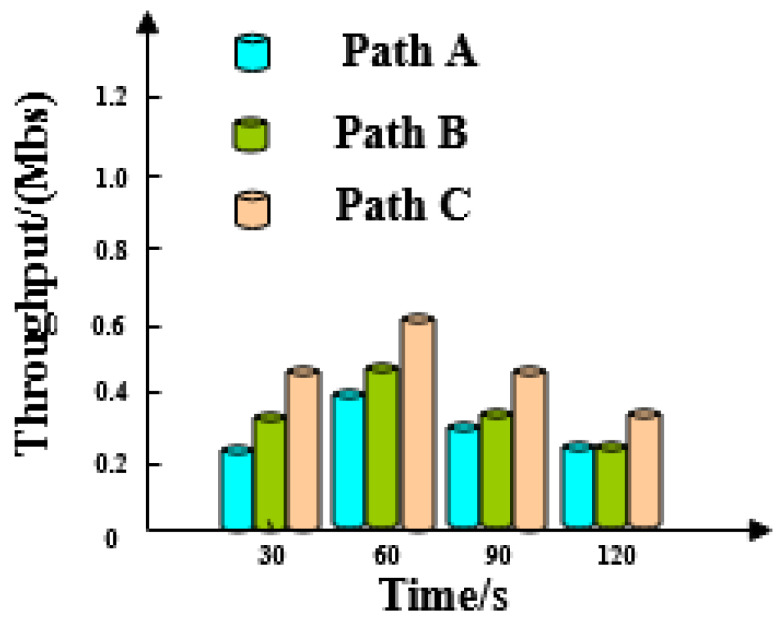
Big data throughput in social networks without our method.

In the process of social network big data scheduling, we define *throughput* to be the amount of data successfully transmitted to facilities such as networks and ports. A higher throughput indicates better performance. The average throughput of each path without the proposed method is shown in Figure 1.

Figure 1 shows that the throughputs of the three paths are increasing in the initial test. When the test is performed for 60 to 120 s, then throughputs of the three paths decrease linearly.

**Figure 2:**
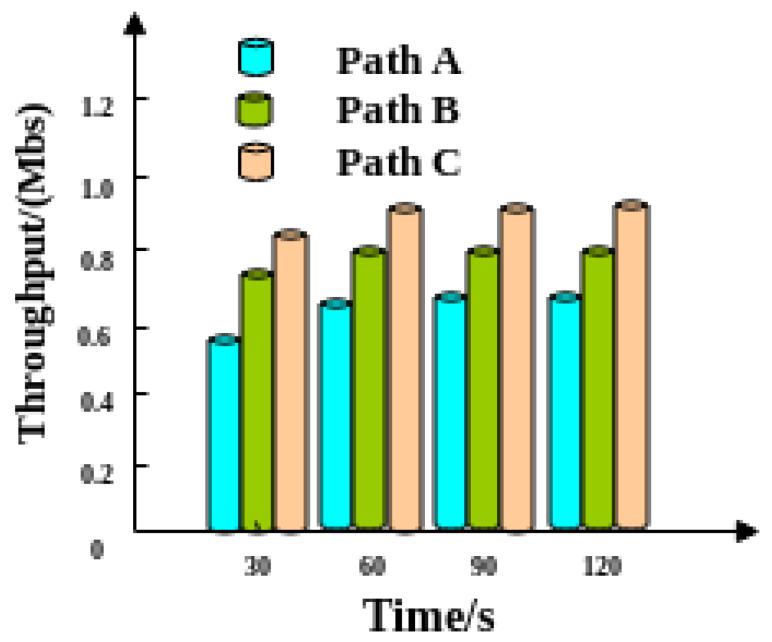
Massive data throughput in social networks using our method.

The average data throughput of each path in the social network after the scheduling is optimized by this method is shown in Figure 2. What we can clearly seen when comparing Figure 1 to Figure 2 is that across the board the throughput increases. Futhermore, in Figure 1 the linear decrease that was evident later in time, is now eliminated using our model and we also see more of a constant throughput as time increases which was an expected result of the optimization.

Figure 2 shows that the throughputs of three paths are stable, and their data throughputs are virtually equal to their bandwidths from about 60 s onwards.

### 4.2. Analysis of Transmission Efficiency

The available bandwidth and delay parameters of each path change faster when scheduling big data in a social network. A massive data scheduling model of social networks should have better adaptability and exhibit excellent data scheduling performance under different network conditions. Therefore, the efficiency and bandwidth consumption of data transmitted by each path, from the perspective of changes in bandwidth, are analyzed before and after the data scheduling model is constructed using this method. The bandwidth consumption of each data transmission path is shown in Table 1. The time taken by each data transmission path to transmit data is shown in Table 2.

Table 1 shows that the transmission bandwidth of path C is bigger the than transmission bandwidth of path B and that the transmission bandwidth of path B is bigger than the transmission bandwidth of path A. Under the above bandwidth, the time consumption of data transmission between three paths is compared. The results are shown in Table 2. In Table 2, after using the method in this paper, although the path bandwidth is constantly changing, data transmission time of path A, path B and path C is stable. Transmission data of path A, path B and path C are approximately 9.3 s, 8.4 s and 8.4 s, respectively.

### 4.3. Performance Comparison

To verify the superiority of this method, its performance in big social network data scheduling optimization experiments is compared against a dynamic organization scheduling method and a greedy algorithm-based scheduling method. These three methods are compared in terms of their performance by the following indicators:

1. Task response time for three methods. This can be understood as the reaction time when a massive data scheduling task in a social network starts and stops.
2. Overall time taken for the task completion of three methods. This can be understood as the time it takes for big data scheduling optimization to start after the first task starts and the last task stops.
3. Efficiency reduction ratio of three methods. This can be understood as a comparison of the response times and actual completion times of the three methods.
4. Optimized social network resource usage rate. This can be understood as a comparison between the effective sharing of a social network resources and the maximum utilization after optimization of three methods.
5. Set different amounts of target data, compare three methods of scheduling optimization with the actual number and analyze the comprehensiveness of scheduling optimization for three methods.
6. Balance. This shows the balance of data nodes.
7. Frequency normalized value. This is used to evaluate the stability of a scheduling optimization method. The smaller the numerical fluctuation, the stronger the stability of a scheduling optimization method.

**Figure 3:**
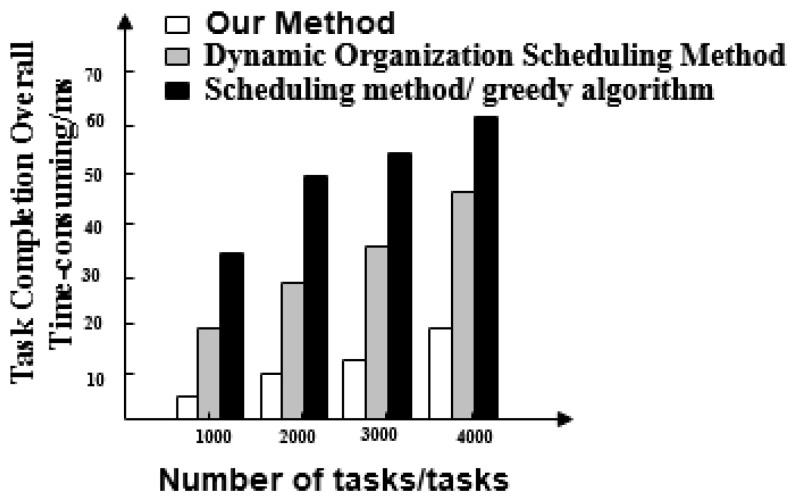
Task response time of the three methods.

The results for scheduling optimization task response time from each of the three methods are shown in Figure 3.

Figure 3 shows that the response time for scheduling optimization tasks is quite different across the three methods. The task response time of the proposed method increases with the number of tasks and the maximum response time is 20 ms. The maximum response times of the dynamic organization scheduling method and the greedy algorithm-based scheduling method are 48 ms and 64 ms, respectively.

**Figure 4:**
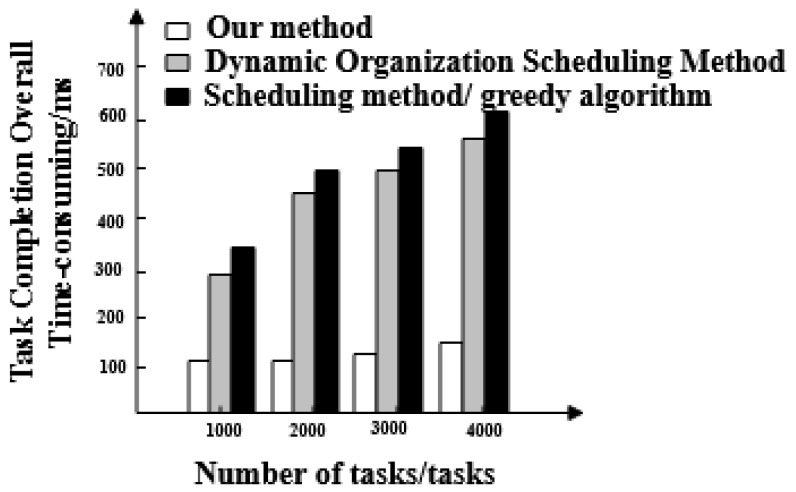
Time-consuming comparison of the overall task completion of the three methods.

Time-consuming comparison results of three methods for scheduling optimization tasks are shown in Figure 4.

Figure 4 shows that overall completion time of the classification optimization scheduling task for the proposed method is less than 200 ms. The overall completion times for the classification optimization scheduling task for dynamic organization scheduling method and the greedy algorithm based on scheduling method increase with the number of tasks. The overall completion time of their tasks also shows a gradual increase and is greater than that of the proposed method.

Efficiency of scheduling optimization for three methods is lower than that of the comparison.

**Figure 5:**
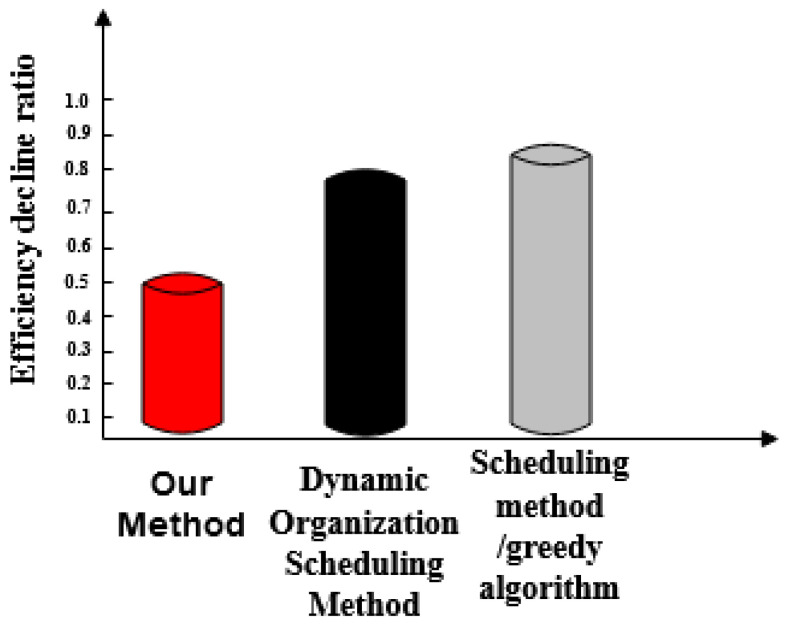
Comparison results of efficiency decline of the three methods.

Figure 5 shows that the efficiency reduction ratio of the proposed method is lower than those of the dynamic organization scheduling method and the greedy algorithm-based scheduling method.

**Figure 6:**
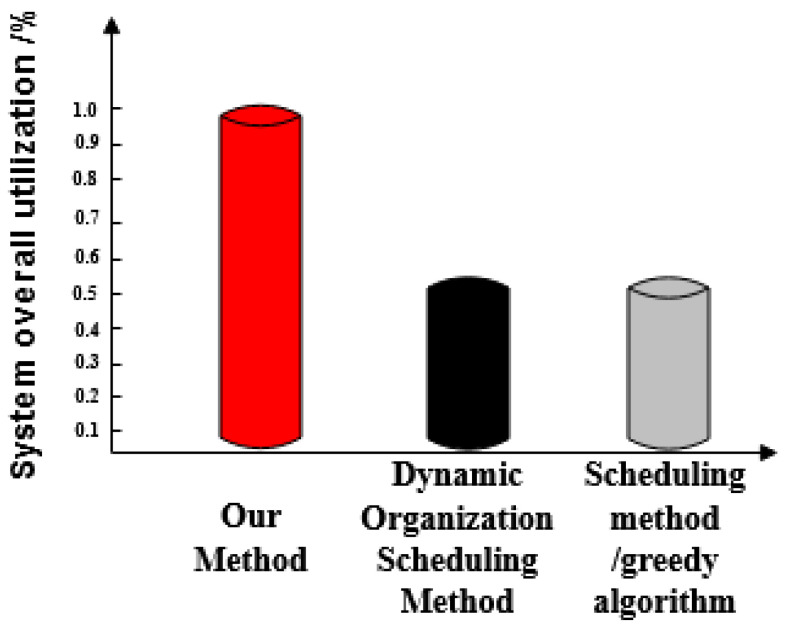
Comparisons of Social Network Resource Utilization Ratio Optimized by Three Methods.

The social network resource utilization rate after three methods of scheduling optimization are compared and comparison results are shown in Figure 6.

Figure 6 shows that the optimized resource usage rate of the proposed method is as high as 100%, which is greater than the resource usage rates of both the dynamic organization scheduling method and the greedy algorithm-based scheduling method.

Comprehensiveness of the scheduling optimization of three methods is shown in Table 3.

Table 3 shows that the maximum difference between the target data volume and the actual data volume is 1 and that its error is small when the proposed method is used. In addition, the maximum difference between the target data volume and the actual data volume when the other two methods are used is greater than that of proposed method.

**Figure 7:**
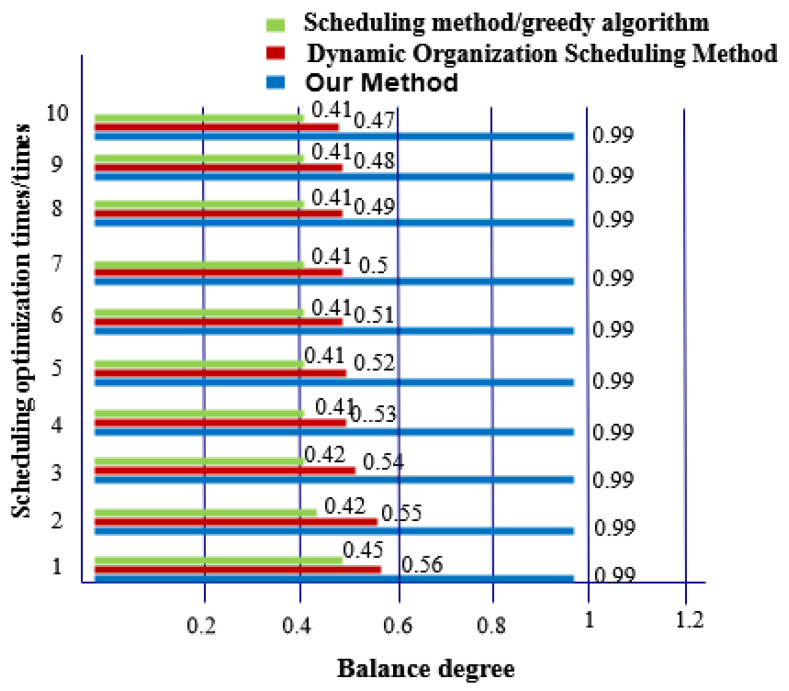
Comparison of Balance Degree of Big Data in Social Networks after Three Methods of Optimizing Scheduling.

The balance of massive data in social networks after three methods are optimized for scheduling are compared and comparison results are shown in Figure 7.

Figure 7 shows that there is a large gap in the balance of big data in social networks after three methods are optimized for scheduling. After the proposed method is optimized, the balance of massive data in a social network is as high as 0.99, which indicates that there is only 0.01 probability of conflict between data. The other two methods only achieve balance degrees not greater than 0.6.

**Figure 8:**
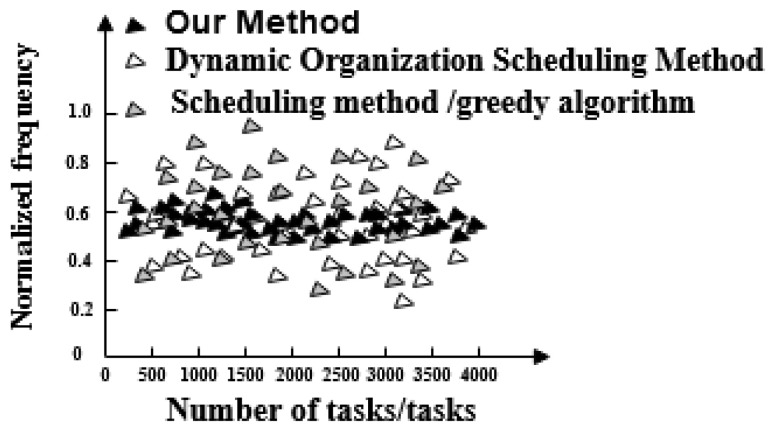
Normalized Frequency Value of Massive Data in Social Networks Scheduled by Three Optimal Methods.

Distribution of the normalized frequency values when three methods optimize massive data of a social network is shown in Figure 8.

Figure 8 shows that the frequency of the scheduling model optimized by the proposed method is between 0.5 and 0.7 and its fluctuation is small. Meanwhile, the frequency of social network data scheduling model optimized by the dynamic organization scheduling method fluctuates between 0.2 and 0.9 and the upper and lower fluctuations are large. The frequency of social network data scheduling model optimized by the greedy algorithm-based scheduling method fluctuates between 0.28 and 0.95, exhibiting a high fluctuation range. Therefore, the social network massive data scheduling model optimized by the proposed method has strong stability and can be used for big data scheduling and optimization in actual social networks.

## 5. Discussion

The results of three path throughput tests are analyzed before using the optimization method in this paper. Experimental results show that throughputs of three paths are increasing at the initial stage of the test but decrease linearly from 60 s to 120 s. Many out-of-order packets exist in the network receiving buffer, occupying a large amount of receiving buffer space and causing a linear reduction in the amount of transmitted data. The throughputs of the three paths after optimization are stable when the test proceeds to about 60 s and the throughput is basically equal to the bandwidth. This phenomenon shows that the maximum possible throughput is achieved after optimization by this method and that bandwidth utilization efficiency is superior.

The three paths satisfy the condition that path C’s transmission bandwidth > path B’s transmission bandwidth > path A’s transmission bandwidth. Under this constraint, the time consumption of data transmission for each of the three paths is compared. It can be seen that after optimization using the proposed method, although the path bandwidth is constantly changing, the data transmission times for the three paths are basically stable. Path A takes approximately 9.3 s to transmit data, Path B takes approximately 8.4 s to transmit data and Path C takes approximately 8.4 s to transmit data. The data shows that massive data scheduling model of a social network optimized by this method adapts to changes in bandwidth. Its data scheduling time is stable, which demonstrates its superior data transmission efficiency.

We compare the performance of the three methods in detail:

1. The three methods have a large difference in scheduling response time. The task response time of the proposed method increases with the number of tasks and the maximum response time is 20 ms. The maximum time-consuming response time of dynamic organization scheduling method and greedy algorithm-based scheduling method is 48 ms and 64 ms, respectively. The task response time of the proposed method is the shortest and tasks are executed quickly.
2. The overall completion time of each task under the proposed method is less than 200 ms. Overall, tasks scheduled under the dynamic organization scheduling method and the greedy algorithm based scheduling method take more time as the number of tasks increases. Their task completion time also gradually increases and takes longer than with the proposed method. The overall completion time of tasks by the proposed method is the shortest of the three.
3. The efficiency reduction ratio of the proposed method is lower than that of the dynamic organization scheduling method and the greedy algorithm based scheduling method. The proposed method exhibits the same efficiency and high stability throughout.
4. The optimized social network resource usage rate under the proposed method is as high as 100%, which is greater than the resource usage rate of the dynamic organization scheduling method and the greedy algorithm-based scheduling method. This demonstrates that there is no redundant data in big data optimized by the proposed method and its availability is high.
5. When the proposed method optimizes big data scheduling in social networks, the maximum difference between the target data volume and the actual data volume is one and its error is small. For the other two methods, the maximum difference between the target data volume and the actual data volume is greater than the proposed method. It can be seen that the optimal scheduling of the proposed method is more comprehensive.
6. There is a big gap in the balance of big data in social networks after the three methods are used. The balance of big data in a social network after scheduling optimization by the proposed method is as high as 0.99, indicating that the probability of conflict between data is only 0.01. After scheduling optimization by the other two methods, the balance of big data in social networks is not more than 0.6, the conflict between data is large and the scheduling optimization process is hindered.
7. The frequency of the scheduling model optimized by the proposed method is between 0.5 and 0.7 and its fluctuation is small. It has the advantage of high stability compared with the other two methods.

In summary, the scheduling optimization effect of this method is significantly better than the dynamic organization scheduling method and the greedy algorithm based scheduling method. This is because the constraints that can be scheduled for big data cycle tasks in social networks [29,30] are analyzed and the distribution function without periodic tasks, is computed using our proposed method. On this basis, the task processor is prompted to follow classification criteria for the task. A flexible transformation of task assignments has been implemented. The product of each scheduled task response resource level and the minimum execution time is calculated. Therefore, the optimization of big data scheduling in social networks is completed by optimizing for the minimum of this product [31,32,33].

## 6. Conclusions

Big data scheduling models of social networks are optimized by our proposed method. The optimization process is as follows—firstly, a big data scheduling model is built. Secondly, the model is optimized to realize optimal scheduling. The social network big data scheduling model constructed in this paper considers the task amount of data communication during target data transmission. Construction of a big data scheduling model based on the amount of tasks effectively solves the problem of conflicting target data in the same data node. Before the method optimizes big data, the necessary conditions for the periodic task schedule ability of social network big data and the calculation of aperiodic task distribution function are analyzed. Experimental results show that the proposed method performs better than a dynamic organization scheduling method and a greedy algorithm-based scheduling method. It adapts to different network environments and solves the problem of load balancing in a short time. In addition, data scheduling and transmission are completed quickly. It provides an effective means for reasonable and efficient scheduling of big data in social networks.

## Figures and Tables

**Table 1:** Data transmission bandwidth for each path.

Task Ordinal Oumber	Bandwidth/(Mb/s)		
	Path A	Path B	Path C
1	0	1.6	2.4
2	0.1	1.5	2.3
3	0.2	1.4	2.2
4	0.3	1.3	2.1
5	0.4	1.2	2.0
6	0.5	1.1	1.9
7	0.6	1.0	1.8
8	0.7	0.9	1.7
9	0.8	0.8	1.6

**Table 2:** Data transmission time comparison results/s.

Task Ordinal Number	Path A		Path B		Path C	
	Before Adopting This Method	After Adopting the Method Presented in This Paper	Before Adopting This Method	After Adopting the Method Presented in This Paper	Before Adopting This Method	After Adopting the Method Presented in This Paper
1	11.89	9.45	12.56	8.56	13.25	8.25
2	35.26	9.56	13.25	8.52	22.54	8.45
3	23.56	9.25	18.25	8.54	20.32	8.25
4	18.22	9.54	16.26	8.26	15.26	8.26
5	14.24	9.24	15.25	8.15	14.23	8.14
6	13.58	9.25	12.33	8.75	12.35	8.75
7	11.95	9.36	13.44	8.49	11.25	8.76
8	11.42	9.37	12.65	8.29	11.04	8.29
9	11.25	9.38	12.47	8.47	11.07	8.61

**Table 3:** Comprehensive scheduling optimization of 3 methods.

Scheduling Optimization Times/Times	Target Data Volume/Number	Article Method	Dynamic Organization Scheduling Method	Scheduling Method Based on Greedy Algorithm
1	500	500	489	491
2	1000	999	989	988
3	1500	1499	1489	1487
4	2000	1999	1991	1989
5	2500	2499	2488	2476
6	3000	2999	2997	2967
7	3500	3499	3498	3467
8	4000	3999	3989	3943
9	4500	4499	4478	4456
10	5000	4999	4967	4934
